# Supplementary figures and images for: Efficacy and indications of tonsillectomy in patients with IgA nephropathy: a retrospective study
Source: PeerJ. 2022 Dec 5;10:e14481. doi: 10.7717/peerj.14481 (PMC9745907; doi:10.7717/peerj.14481)

M0

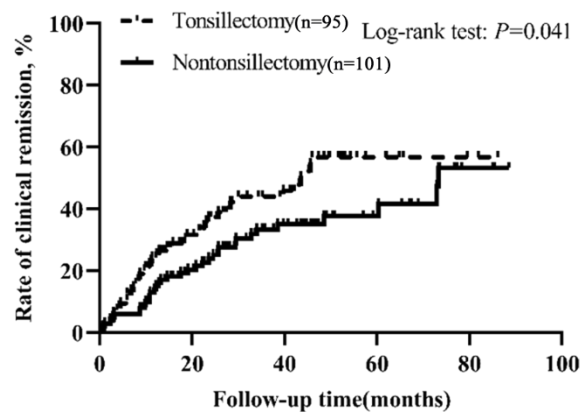

M1

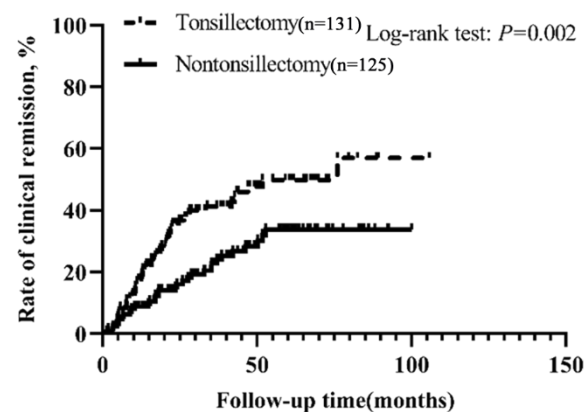

E0

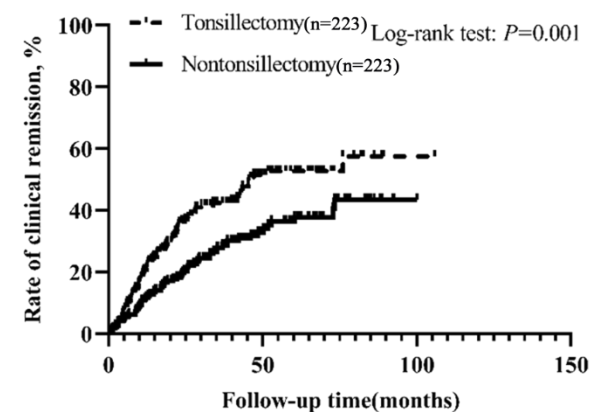

S0

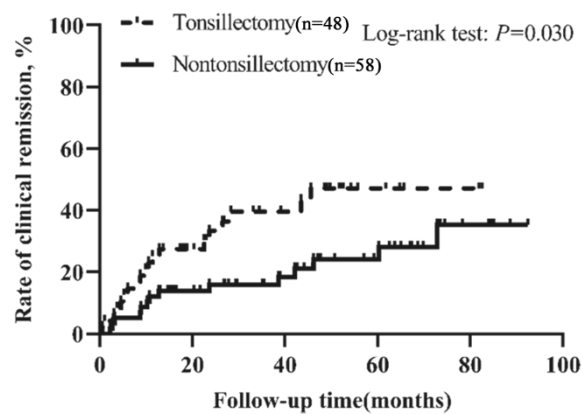

S1

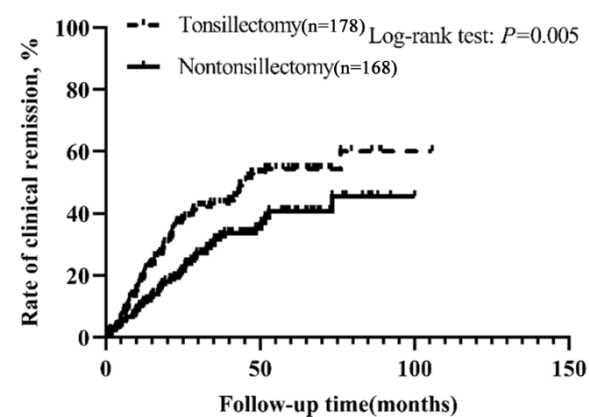

T0

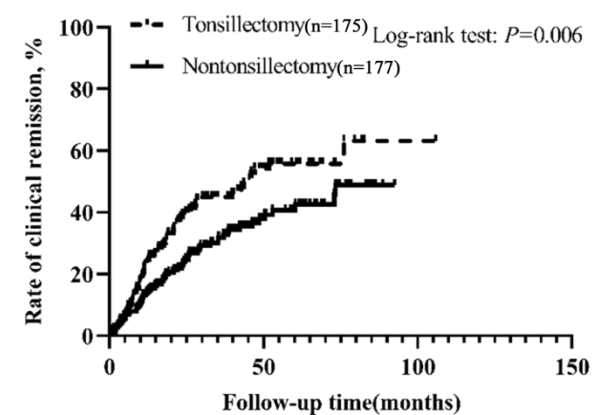

T1

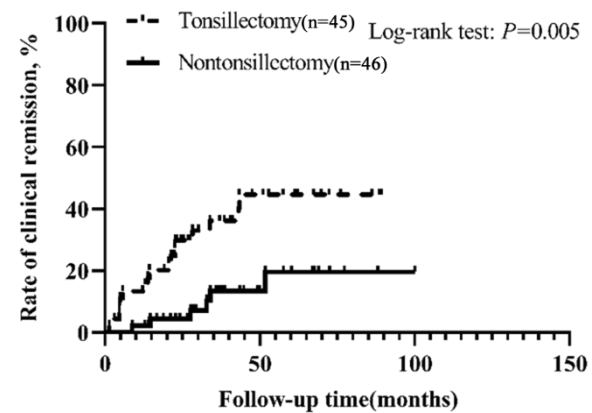

C0

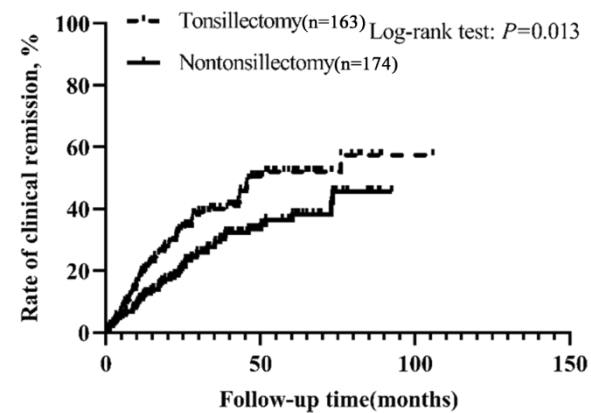

C1

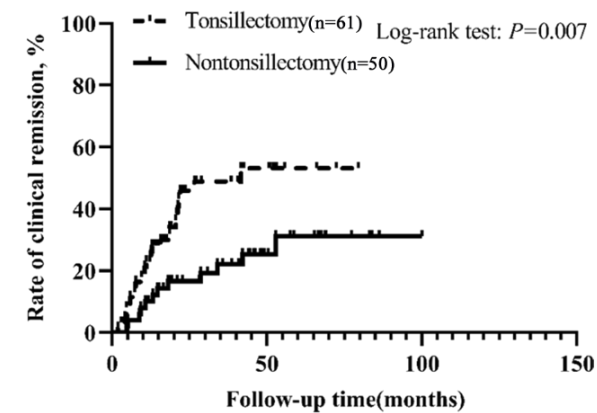

Supplement: Supplemental Information 1 [file peerj-10-14481-s001.pdf]

M0

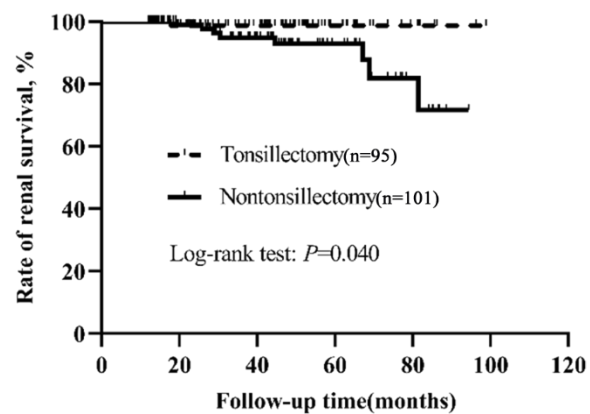

M1

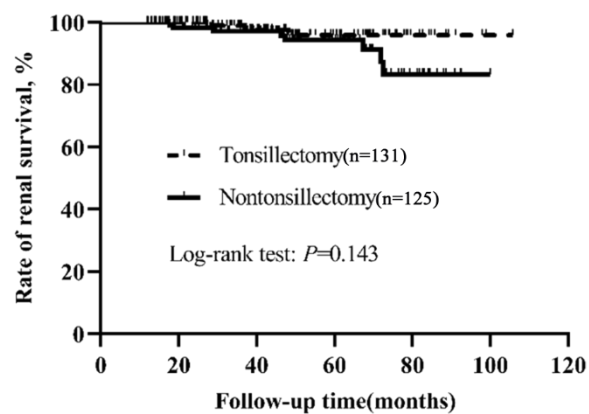

E0

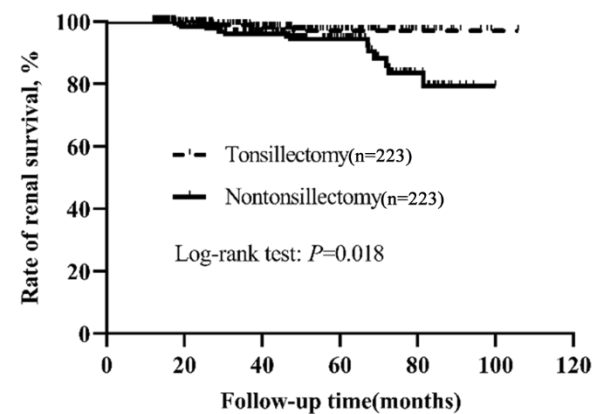

S0

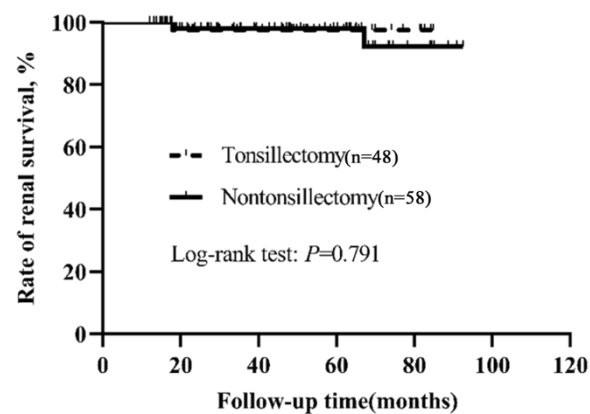

S1

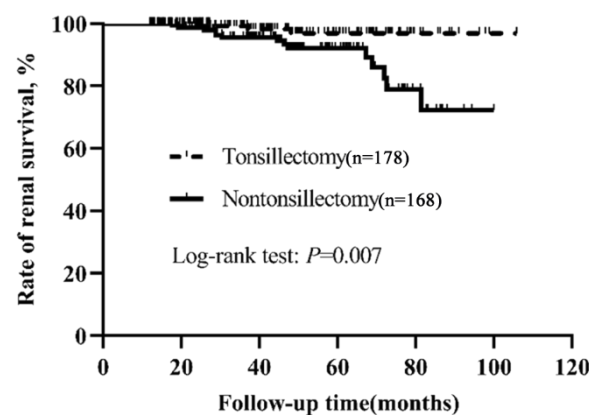

T0

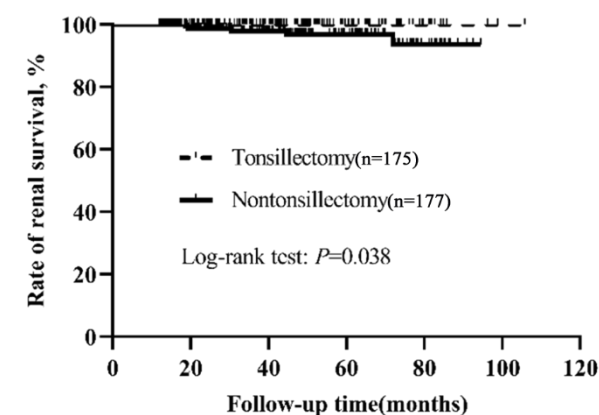

T1

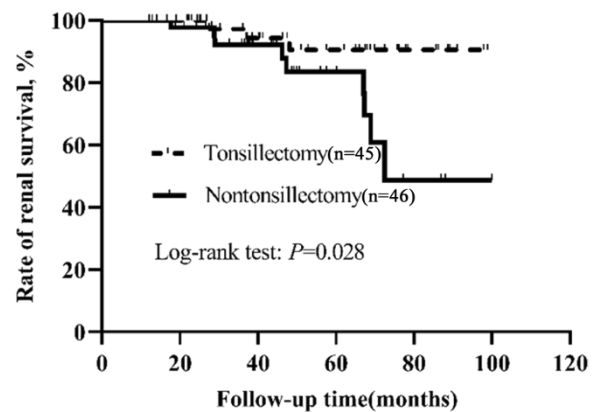

C0

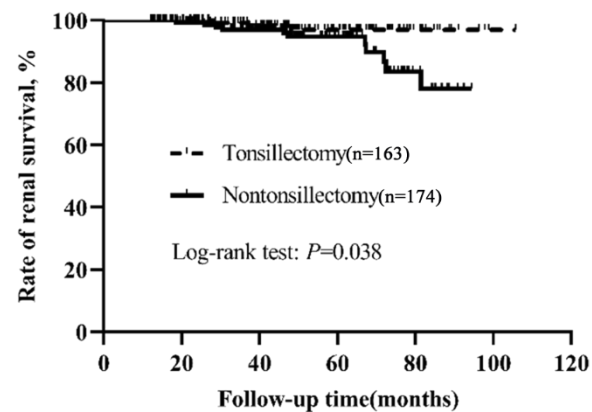

C1

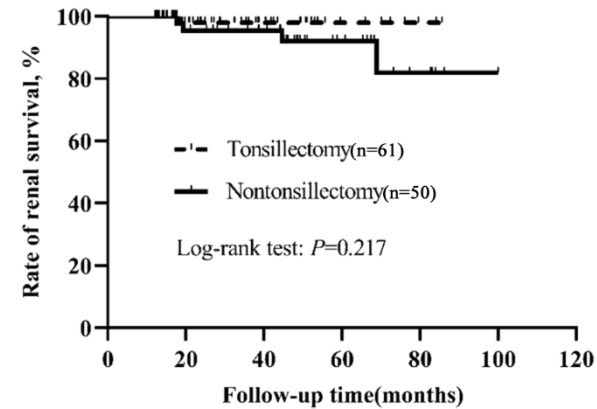

Supplement: Supplemental Information 2 [file peerj-10-14481-s002.pdf]
