# Supplementary material for: Efficacy and indications of tonsillectomy in patients with IgA nephropathy: a retrospective study
Source: PeerJ. 2022 Dec 5;10:e14481. doi: 10.7717/peerj.14481 (PMC9745907; doi:10.7717/peerj.14481)
Supplement: Supplemental Information 3 [file peerj-10-14481-s003.pdf]

Table S1:

Comparison of hematuria and proteinuria between the tonsillectomy and nontonsillectomy groups before and after treatment.

	Hematuria			Proteinuria		
	Tonsillectomy	Nontonsillectomy	*P*-value	Tonsillectomy	Nontonsillectomy	*P*-value
Before treatment	5/17/35/100/69	4/25/39/97/61	0.665	19/19/114/55/19	22/32/111/51/10	0.164
After treatment	58/22/49/66/31	41/14/51/68/52	0.039*	129/31/35/22/9	106/25/52/37/6	0.031*

Values are expressed as the counts of hematuria score or proteinuria score, 0/0.5/1/2/3.

*Statistically significant
