# Supplementary material for: Efficacy and indications of tonsillectomy in patients with IgA nephropathy: a retrospective study
Source: PeerJ. 2022 Dec 5;10:e14481. doi: 10.7717/peerj.14481 (PMC9745907; doi:10.7717/peerj.14481)
Supplement: Supplemental Information 4 [file peerj-10-14481-s004.pdf]

Table S2：

Comparison of background therapy between the tonsillectomy and nontonsillectomy groups under different proteinuria grades.

Background therapy	Steroid/ Immunosuppressant			RAS inhibitor		
	Tonsillectomy	Nontonsillectomy	*P*-value	Tonsillectomy	Nontonsillectomy	*P*-value
≤1g/24h	84/154	88/166	0.783	108/154	113/166	0.691
>1g/24h	45/72	35/60	0.626	54/72	41/60	0.396
