# Supplementary material for: Efficacy and indications of tonsillectomy in patients with IgA nephropathy: a retrospective study
Source: PeerJ. 2022 Dec 5;10:e14481. doi: 10.7717/peerj.14481 (PMC9745907; doi:10.7717/peerj.14481)
Supplement: Supplemental Information 5 [file peerj-10-14481-s005.pdf]

Table S3:

Comparison of background therapy between the tonsillectomy and nontonsillectomy groups under different MEST-C scores.

Background therapy	Steroid/ Immunosuppressant			RAS inhibitor		
	Tonsillectomy	Nontonsillectomy	*P*-value	Tonsillectomy	Nontonsillectomy	*P*-value
M0	46/95	53/101	0.570	63/95	64/101	0.666
M1	83/131	70/125	0.230	99/131	90/125	0.516
E0	127/223	120/223	0.505	160/223	152/223	0.409
E1	2/3	3/3	0.273	2/3	2/3	1.000
S0	22/48	29/58	0.669	27/48	33/58	0.947
S1	107/178	94/168	0.433	135/178	121/168	0.418
T0	95/175	92/177	0.664	121/175	110/177	0.167
T1	30/45	29/46	0.717	38/45	41/46	0.509
T2	4/6	2/3	1.000	3/6	3/3	0.134
C0	93/163	89/174	0.277	117/163	111/174	0.117
C1	35/61	34/50	0.251	43/61	41/50	0.160
C2	1/2	0/2	0.248	2/2	2/2	1.000
